# Using MRI to guide surgical strategy for perianal fistulas: from conventional acquisition to 3D modeling

**DOI:** 10.1590/0100-3984.2025.0064-en

**Published:** 2026-02-26

**Authors:** Priscilla Ornellas Neves, André Araújo de Medeiros Silva

**Affiliations:** 1 Department of Radiology, Brasilia University Hospital, Brasília, DF, Brazil.; 2 Hospital DF Star/Rede Dor, Brasília, DF, Brazil.; 3 Department of Colorectal Surgery. School of Medicine, University of Brasília, Brasília, DF, Brazil.; 4 State Department of Health of the Federal District, Brasília, DF, Brazil.

**Keywords:** Rectal fistula/diagnostic imaging, Anal canal/surgery, Magnetic resonance imaging, Imaging, three-dimensional, Colorectal surgery, Fístula retal/diagnóstico por imagem, Canal anal/cirurgia, Imageamento por ressonância magnética, Imageamento tridimensional, Cirurgia colorretal

## Abstract

Magnetic resonance imaging (MRI) is the gold-standard imaging modality for evaluating perianal fistulas, playing a pivotal role in characterizing fistula anatomy and guiding optimal surgical strategies. Appropriate preoperative MRI assessment can significantly reduce recurrence rates and minimize the risk of complications, such as fecal incontinence. This article reviews the most relevant information that an MRI report should contain to aid effective surgical decision-making for perianal fistulas. These features influence therapeutic choices, including decisions regarding the suitability of techniques like fistulotomy, ligation of the intersphincteric fistula tract (LIFT), video-assisted anal fistula treatment (VAAFT), fistula-tract laser closure (FiLaC), and advancement flaps. The emerging role of three-dimensional (3D) modeling from MRI data is also discussed, highlighting its potential to enhance surgeons’ spatial understanding of complex fistulous anatomy, thereby improving surgical planning, reducing operative time, and ultimately improving outcomes. However, the generation of accurate 3D models depends on meticulous image segmentation and interpretation by experienced radiologists. Future research directions, including the integration of 3D models with intraoperative navigation and standardized assessment of inflammatory activity, are also addressed in this review.

## Abstract (pt)

A ressonância magnética (RM) é o método de imagem padrão-ouro na avaliação das fístulas perianais, fundamental na caracterização anatômica dos trajetos fistulosos e na orientação das estratégias cirúrgicas. A avaliação préoperatória por RM reduz taxas de recidiva e o risco de complicações, como incontinência fecal. Este artigo revisa os principais elementos que devem compor o laudo de RM para otimizar o planejamento cirúrgico. Essas informações impactam diretamente na escolha terapêutica, incluindo técnicas como fistulotomia, ligadura do trajeto interesfinctérico da fístula (LIFT), tratamento de fístula anal assistido por vídeo (VAAFT), fechamento do trajeto fistuloso com laser (FiLaC) ou retalhos de avanço. Discute-se ainda o papel emergente da reconstrução de modelo tridimensional (3D) a partir de dados de RM, destacando seu potencial em aprimorar a compreensão espacial da anatomia complexa das fístulas, favorecer o planejamento cirúrgico, reduzir o tempo operatório e melhorar os resultados clínicos. No entanto, a geração de modelos 3D precisos requer segmentação meticulosa e interpretação criteriosa das imagens por radiologistas experientes. São também abordadas perspectivas futuras, como a integração de modelos 3D com sistemas de navegação intraoperatória e a padronização da avaliação da atividade inflamatória.

## INTRODUCTION

Perianal fistulas represent a challenging condition in clinical practice because of their anatomical complexity and high recurrence rates. Accurate preoperative assessment is essential to determine the most appropriate surgical technique and to reduce the risk of postoperative complications, including sphincter damage and incontinence^**(^1^)**^. Magnetic resonance imaging (MRI) is the gold standard imaging method for evaluating perianal fistulas, enabling the characterization of fistulas and informing decisions regarding the best treatment strategy. In addition, MRI provides detailed visualization of the fistulous tract, internal/external openings, secondary extensions, and associated collections—factors that directly influence surgical planning^**(^1,2^)**^. Furthermore, the integration of three-dimensional (3D) models based on MRI data has emerged as a promising tool in preoperative evaluation, offering improved anatomical understanding for surgeons and radiologists. These models may enhance multidisciplinary decision making, particularly in cases of complex or recurrent fistulas^**(^3,4^)**^.

The objective of this article is to review the most relevant information in the MRI report of anorectal fistula in the scenarios of conventional and minimally invasive surgical techniques, on the basis of the most recent guidelines, and to discuss the role of additional tools for treatment planning, such as 3D modeling. This was a narrative review based on a structured search of PubMed and Google Scholar (2000–2024), using the following search terms: “perianal fistula”; “magnetic resonance imaging”; “surgical planning”; “3D modeling”; and “colorectal surgery”. We included studies and consensus statements addressing the role of MRI in the preoperative assessment or surgical decision making for anal fistulas, excluding case reports and studies unrelated to MRI or surgical planning.

### Background

Anal fistula is a condition characterized by nonphysiological communication between the anus or rectum and the skin, usually in the perianal or perineal region.^**(^2^)**^ Perianal fistulas affect approximately two in every 10,000 people annually; most are of cryptoglandular origin, presumably resulting from a chronic infection related to a perianal abscess^**(^5^)**^. The second most common cause is Crohn’s disease, which accounts for approximately a third of all cases. Additional etiologies include trauma, other infections, and pelvic neoplasms^**(^6,7^)**^. Fistulas can be categorized as simple or complex, which guides the treatment approach^**(^8,9^)**^. Complex fistulas are characterized as follows^**(^6^)**^: trans-sphincteric, with involvement of more than 30% of the external sphincter; suprasphincteric; extrasphincteric; horseshoe-shaped; recurrent; those in an anterior position in women; and those associated with inflammatory bowel disease; pelvic radiation, or malignancy. The most effective treatment for anorectal fistula of cryptoglandular origin is surgery, which remains a challenge for colorectal surgeons^**(^1^)**^. Knowledge of the morphology of the fistula and its relationship with the sphincter complex are crucial for determining the viability of any surgical procedure. The best surgical strategy should offer the best chance of cure with the lowest risk of recurrence and an acceptable risk of loss of continence^**(^5^)**^. There are several preoperative, intraoperative, and postoperative risk factors for fistula recurrence, which occurs in 7–50% of cases^**(^3^)**^, the most relevant being the complexity of the fistula and its inadequate characterization, such as failure to identify tracts and inability to locate the internal opening^**(^2,10^)**^. Imaging examinations are used in order to facilitate the adequate identification and morphological characterization of the fistula, thereby informing the surgical strategy to be employed^**(^2^)**^.

**Figure 1:**
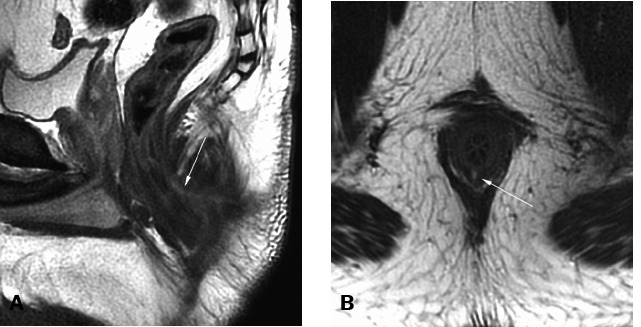
24-year-old male. Simple posterior intersphincteric fistula, with its internal opening at the mid-anal canal, without collections or secondary tracts. Fistulotomy is a safe procedure for this type of fistula. **A.** Sagittal T2-weighted image showing the posterior intersphincteric fistula (white arrow). **B.** Axial T2-weighted image showing the inflammatory activity demonstrated by high T2 signal (white arrow).

**Figure 2:**
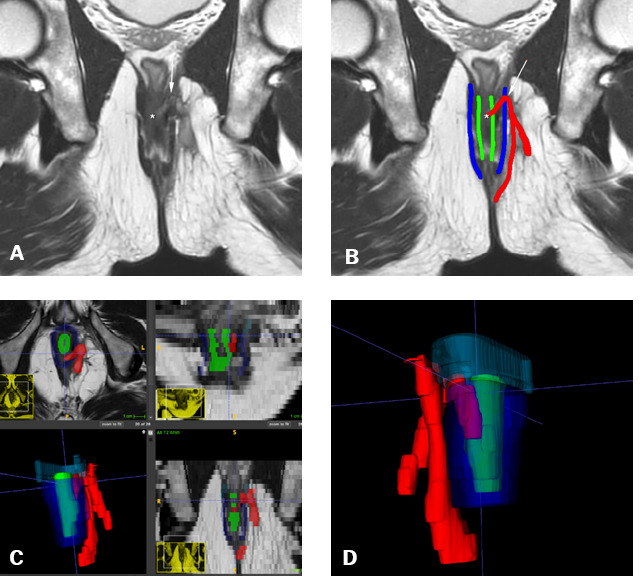
59-year-old male. High posterior transsphincteric fistula. The ascending tract within the intersphincteric space transfixes the puborectalis muscle, potentially misclassified as a lower fistula on anal inspection. Complex bifid extension to the left ischioanal fossa. **A,B:** Oblique coronal T2-weighted MRI sequence showing the internal opening (*) and ascending transsphincteric tract within the intersphincteric space (white arrow; red line in **B**), with the EAS in blue and the IAS in green. **C:** Image segmentation of the fistulous tract (red), IAS (green), and EAS (dark blue), performed on axial T2-weighted turbo spin-echo images. **D:** 3D model in a posterior view, showing the internal opening and the ascending tract crossing the puborectalis muscle (light blue).

**Figure 3:**
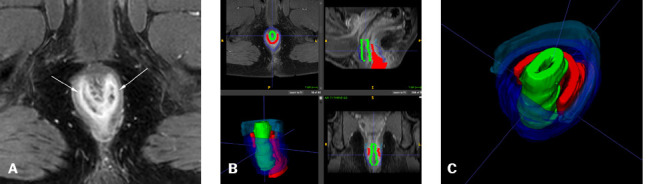
29-year-old male. Posterior intersphincteric horseshoe fistula at the mid-anal canal, extending between 1 and 11 o’clock and perianal abscess. Horseshoe tracts are typically unsuitable for minimally invasive techniques such as LIFT, VAAFT, and FiLaC. **A:** Axial contrast-enhanced image showing the horseshoe fistula (white arrow). **B:** Image segmentation performed on axial contrast-enhanced T1-weighted images. Note that the intersphincteric tract (red) terminates in an abscess in the posterior perianal region. **C:** 3D model in a top view, showing the intersphincteric horseshoe fistulous tract crossing the midline.

**Figure 4:**
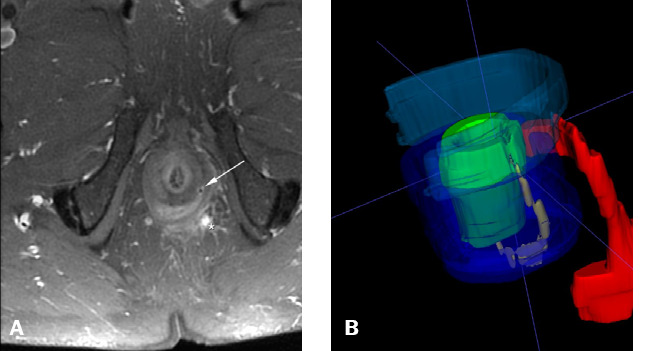
64-year-old male. High posterior transsphinctericfistula with active inflammatory signs and a predominance of granulation tissue. Left intersphincteric tract with seton placement, without inflammatory activity or abscess. Transsphinctericfistula treated with VAAFT, with signs of healing on follow-up. **A.** Axial contrast-enhanced T1-weighted image: seton (white arrow) and transsphincterictract (*). **B.** 3D model in a lateral view, showing the intersphincteric seton located along the left lateral wall (yellow) and the active fistulous tract with posterior origin (red), not drained by the seton, together with a collection in the posterior perianal margin.

**Figure 5:**
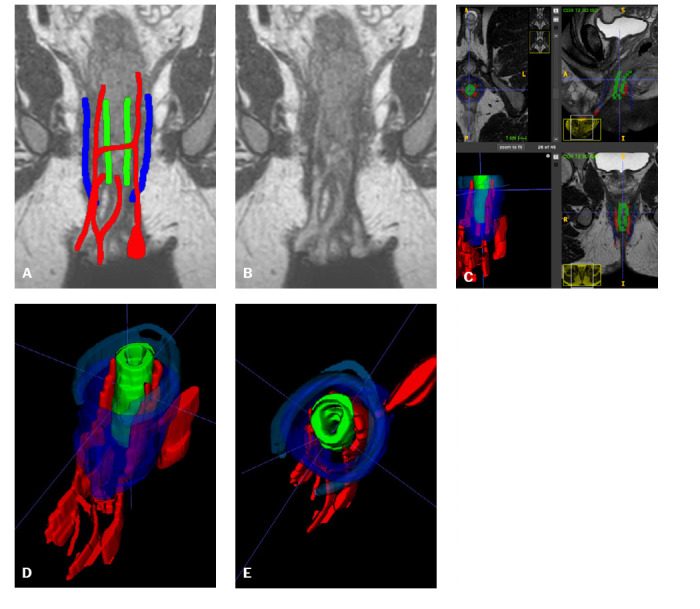
24-year-old male. Crohn’s disease with perianal involvement. Complex anal fistula with multiple origins, including four at the level of the puborectalis muscle. A posterior intersphincteric horseshoe tract communicates with these fistulas at the mid-anal canal. There is a collection extending into the left ischioanal fossa, as well as multiple anterior tracts reaching the scrotal margin. Initial management included drainage and seton placement, followed by biologic therapy. **A,B:** Coronal 3D isotropic images showing a complex fistula (red lines in A; EAS in blue and IAS in green). **C:** Image segmentation on the coronal isotropic T2-weighted volumetric sequence. The fistulous tract with multiple origins was segmented in the axial plane. **D:** 3D model in a frontolateral view, showing anterior extension of multiple tracts. **E:** 3D model in a superior (top) view, showing the posterior left tract forming a collection in the topography of the ischioanal fossa.

Various techniques can be used for the morphological evaluation of the fistula and its relationships with pelvic and perianal structures, including X-ray fistulography, computed tomography, endorectal or transrectal ultrasound, and MRI^**(^11^)**^. Surgeons have access to 3D reconstructed endorectal ultrasound as a tool that can be used in the office and in outpatient settings, making it helpful in characterizing fistulas and anorectal abscesses, identifying the internal opening, and determining the main tract of a fistula. Among the various imaging modalities, MRI is the method of choice for studying anorectal fistulas, because it is superior for characterizing secondary tracts^**(^2,6^)**^. Evidence from observational studies summarized in a narrative review has shown that MRI-guided surgery can reduce the likelihood of recurrence by approximately 75%, mainly by allowing precise preoperative identification of fistulous tracts and secondary extensions that might otherwise go undetected^**(^5^)**^. Prospective observational studies suggest that this imaging method, used preoperatively, modifies surgical strategy in approximately 10% of patients with primary fistulas and 21% of those with primary, secondary, or Crohn’s disease-related fistulas^**(^1^)**^. The benefits are even greater for sphincter-preserving procedures^**(^5^)**^. Certain aspects of fistula morphology favor certain surgical procedures, whereas other procedures are contraindicated by specific morphological characteristics^**(^7^)**^. Several studies have discussed which are the most relevant characteristics to be described in MRI reports in the evaluation of anorectal fistulas, as well as the best form of presentation, such as structured reporting, to improve understanding on the part of the attending physician^**(^2,5,12^)**^. However, in cases of complex anorectal fistulas, reports can still be challenging to interpret for adequate surgical planning^**(^3^)**^. In recent years, 3D modeling has gained space as a promising tool to aid in surgical planning^**(^3^)**^. Figures 1–5 demonstrate MRI-based strategies in the evaluation of perianal fistulas, highlighting traditional 2D sequences as well as 3D reconstructions.

### Relevant anatomy of the anal canal

The sphincter complex of the anal canal consists of the internal anal sphincter (IAS), which is a smooth muscle, a continuation of the internal circular muscle of the rectum. On T2-weighted imaging, it shows a signal that is hyperintense in comparison with that of the external sphincter. The intersphincteric space contains fat and the longitudinal muscle, separating the sphincters. The external anal sphincter (EAS) is superiorly continuous with the puborectalis muscle, a U-shaped skeletal muscle, which forms a sling passing posterior to the anorectal junction. The puborectalis joins the levator ani superiorly, forming the pelvic floor and inserting into the pelvic wall. The ischioanal fossa is a space containing fat surrounding the sphincter complex, delimited medially and superiorly by the EAS and levator ani. That is the space affected by transsphincteric fistulas^**(^5^)**^.

### Classification of anorectal fistulas

There are several classification systems for anorectal fistulas. The most common are described in simplified form below.

The Parks classification divides anorectal fistulas into intersphincteric, transsphincteric, suprasphincteric, and extrasphincteric, the first two being the most common. Submucosal fistulas are those in which there is no involvement of the sphincter complex in their formation, having a different etiology, and are not included in the original Parks classification^**(^13^)**^.

The St. James’s University Hospital classification is based on MRI assessment, classifying anorectal fistulas into five grades of complexity by attributing predictive value to MRI for postoperative outcomes, considering the presence of secondary tracts and abscesses^**(^14^)**^: grade 1, simple linear intersphincteric fistulization; grade 2, intersphincteric fistulization with abscess or secondary tract; grade 3, transsphincteric fistulization; grade 4, transsphincteric fistulization with abscess or secondary tract within the ischioanal fossa; and grade 5, fistula with supralevator or translevator/extrasphincteric extension.

From a practical point of view, all anal fistula classification systems describe two distinct conditions^**(^6^)**^: simple fistulas, for which fistulotomy is a safe procedure with high cure rates and no postoperative continence problems; and complex fistulas, in which there is significant involvement of the anal sphincter, associated with higher rates of recurrence and risk of incontinence, which should be treated exclusively with sphincter-preserving techniques.

### Surgical techniques

Currently, there is no surgical technique that is universally superior for all fistulas^**(^6^)**^.Among the possible surgical treatments, fistulotomy is the procedure most frequently performed for simple fistulas, which account for approximately 30–50% of cases, with a cure rate of approximately 95% when all tracts are treated. In general, fistulotomy results in a reliable cure and reasonable patient satisfaction when 2 cm of the most cephalic EAS is preserved, and it is generally used in intersphincteric and low transsphincteric fistulas^**(^1,6^)**^. However, fistulotomy carries a high risk of fecal incontinence, especially when performed for complex fistulas^**(^15^)**^.For complex anal fistulas, only sphincter-preserving techniques should be employed. The objective of the surgery is, first, to remove or destroy the fistulous tract while preserving the integrity of the sphincters, and then to identify risk factors for fistula recurrence^**(^6^)**^.

#### Minimally invasive sphincter-preserving surgical techniques for complex fistulas

Minimally invasive surgical procedures, with total preservation of the sphincter musculature and consequent maintenance of its function, have become the method of choice in recent decades for the treatment of complex fistulas. The viability of a sphincter-preserving procedure is, in part, determined by specific anatomical characteristics, such as tortuosity, diameter, and the presence of intersphincteric complexity^**(^5,9^)**^.

- **VAAFT (Video-assisted anal fistula treatment—**First described in 2011, video-assisted anal fistula treatment (VAAFT) consists in using an 8° angled fistuloscope, with an optical and working channel, and irrigation. The operation begins with a diagnostic phase aimed at identifying the internal opening and determining the tract, as well as identifying possible secondary tracts and collections. The fistuloscope is introduced through the internal opening using an electrolyte-free solution, usually 1.5% glycine or 1% mannitol^**(^16^)**^, because of its nonconductive property and transparency, to remove debris and blood^**(^17^)**^, traversing the tract and identifying the internal opening. That is followed by the operative phase, in which the fistulous tract is treated by electrocautery the fistula walls and removal of debris. At the end of the procedure, the internal opening is treated, and the external opening is enlarged for adequate drainage^**(^16,18^)**^.
- **FiLaC (Fistula-tract laser closure —** Also in 2011, fistula-tract laser closure (FiLaC), which involves the use of a radial emitting laser probe aimed at destroying the epithelium and obliterating the tract, was first described as a treatment for perianal fistula. In FiLaC, a probe is inserted through the fistulous tract blindly until it reaches the internal opening. Energy is then applied during continuous retraction of the probe (1 cm/s), until its complete removal. At the end of the procedure, the internal opening is treated by creating a flap or suture^**(^19^–^21^)**^.
- **Transanal opening of the intersphincteric space —** One recent method is transanal opening of the intersphincteric space with secondary healing, which achieves high cure rates (>90%) without significant reports of postoperative incontinence^**(^22^)**^.
- **Over-the-scope clip (OTSC) —** The endoscopic technique known as over-the-scope clip uses a metallic clip to close the internal opening of an anorectal fistula. Cure rates range from 45% to70%. This technique is more effective as an initial therapy, and is less efficient in recurrent cases or inflammatory fistulas^**(^22^)**^.
- **Cutting seton—**The cutting seton technique consists in passing a thread through the fistulous tract, with progressive sectioning of the EAS. Currently, the technique is less recommended because it is associated with high rates of postoperative incontinence (12–53%).
- **Loose seton—**Loose setons, also known as draining setons, are used in order to control perianal sepsis, mainly in acute cases or cases of anal Crohn’s disease. The setons can be left in place for weeks, months, or years, depending on the local clinical evolution of sepsis.
- **Ligation of the intersphincteric fistula tract —** Ligation of the intersphincteric fistula tract (LIFT) is a procedure that involves dissection and ligation of the fistulous tract in the intersphincteric space. It can be used for complex fistulas and for simple low transsphincteric fistulas. The success rate is 60–90%, with low rates of postoperative incontinence.
- **Rectal advancement fiap—**The internal opening of a fistula can be closed by using a mucosal or submucosal flap, known as an advancement flap. Although the success rate ranges from 40% to 85%, the use of this technique can lead to some degree of postoperative incontinence. It can be used in combination with other techniques, such as LIFT.
- **Stem cell therapy for anal fistula —** Stem cell therapy is a high-cost treatment, indicated for carefully selected patients with refractory perianal fistulizing Crohn’s disease who have not responded to other, conventional therapies. There have been only a few reports of its use, with a limited number of patients.

These techniques reflect different approaches to preserve sphincteric integrity and avoid postoperative complications, with the choice of method being influenced by the anatomical complexity and etiology of the fistula, preferably assessed by imaging examinations such as MRI. Currently, there is no consensus on the absolute superiority of a specific technique; individualized treatment, tailored to the complexity and patient profile, is recommended, and one method may be combined with another^**(^22^)**^. Chart 1 summarizes the key anatomical characteristics of perianal fistulas and their relevance in determining the suitability of various surgical techniques.

**Chart 1:** Characteristics of perianal fistulas and radiological predictors of the suitability of surgical techniques.

Characteristic	Radiological considerations	Suitable techniques	Unsuitable techniques
Tract height	Distance from tract to anorectal junction; amount of EAS/IAS above the fistula	Fistulotomy (if low); FiLaC (if transsphincteric)	Fistulotomy (if high); LIFT (if very high intersphincteric)
Tract direction (obliquity)	Oblique cephalic tracts cross more sphincter and may affect continence	FiLaC; VAAFT	Fistulotomy if significant cephalic obliquity
Internal opening position	High openings near the anorectal junction, which are harder to access or close	VAAFT; FiLaC (some cases); RAF	LIFT (if high); plug (if deep or inaccessible)
Tract complexity (branches/extensions)	Horseshoe, supralevator, or intersphincteric extensions on MRI	RAF; VAAFT (selective); drainage with setons	FiLaC; plug; LIFT
Tract diameter	Narrow: may hinder VAAFT/ FiLaC access; Wide: risk of failure with laser/plug	FiLaC (if 3-5 mm); VAAFT (if > 3.3 mm)	FiLaC (for cavities); plug (for very wide tracts)
Intersphincteric space involvement	Oblique, wide tracts, or horseshoe extensions, which complicate ligation	LIFT (if straight, narrow, and intersphincteric)	LIFT (if horseshoe or with IAS defect)
Abscesses/collections	Hyperintense foci on T2/STIR MRI; require complete drainage	VAAFT (visualization); setons prior to surgery	Plug or FiLaC if collections untreated
IAS integrity	Defects or thinning, which may predict incontinence risk or flap failure	VAAFT; RAF (if IAS intact)	LIFT (if IAS disrupted); RAF (if damaged)
Previous surgery/ scarring	Altered anatomy on MRI, which affects flap mobility and sphincter function	Partial-thickness RAF; VAAFT	LIFT (if space distorted); full-thickness RAF
Inflammatory activity (Crohn's)	Proctitis or active inflammation on MRI, either of which impedes flap healing	Seton; VAAFT (for symptom control)	RAF (in active proctitis); plug

RAF, rectal advancement flap; STIR, short-tau inversion recovery.

### Descriptors in the MRI report

To maximize clinical utility, the MRI report must accurately describe the clinically relevant characteristics, summarized in Chart 2.

**Chart 2:** Primary descriptors in anorectal fistula MRI.

Descriptor category	Essential features to report
Classification	Relationship with sphincteric complex: intersphincteric, transsphincteric, extrasphincteric, or suprasphincteric
Internal opening	Radial (clock-face) position; height (upper/middle/lower third of the anal canal); number and size
Tract pathway	Direction (cephalic/caudal); angulation; extensions if present (horseshoe, blind)
External opening	Radial position; anatomical site (e.g., gluteal, labial, perianal skin, etc.)
Collections/abscesses	Presence; diameter (<10mm = small, 11-20mm = medium, and >20mm = large); location; and connection to primary tract
Fistula activity	Active vs. inactive (based on T2/ STIR hyperintensity); and presence of granulation or fibrotic tissue

STIR, short-tau inversion recovery. Note: Data presented in this table were obtained in part from Halligan^**(^2^)**^, Iqbal et al.^**(^5^)**^, and Yushkevich et al.^**(^29^)**^.

#### Internal opening

The height of the internal opening in the anal canal and its radial location are well established as fundamental characteristics in the MRI report^**(^2^)**^, as are other features of the opening:

- **Height—**Describe this using the descriptors upper, middle, or lower third of the anal canal, based on the measurement of the striated musculature, including the puborectalis, and mention the plane in which it is best characterized (sagittal or coronal). High fistulas, such as extrasphincteric ones, are difficult to access for FiLaC^**(^5^)**^.
- **Radial location—**This should be reported according to the anal clock, a nomenclature familiar to clinicians, radiologists, and surgeons in the field. The quadrant, such as right anterior, left posterior, etc., can be included in the description^**(^2,5^)**^.
- **Diameter—**This parameter may be critical in specific scenarios, particularly in the planning of minimally invasive surgical procedures. A internal opening that is wide (defined in one study as a diameter greater than 5 mm) may predict greater difficulty in conventional closure of the orifice. According to a multidisciplinary expert consensus process^**(^23^)**^, measurement of the internal opening diameter on MRI shows moderate interobserver agreement and should therefore be interpreted in conjunction with the surgical findings. Despite variability, this consensus and the narrative review authored by Iqbal et al.^**(^5^)**^ both emphasize that a wide internal opening remains clinically relevant, especially for patients in whom sphincter-preserving techniques such as VAAFT and FiLaC are indicated, as well as for determining the flap size and tension required for advancement flap procedures^**(^5,23^)**^.

#### Tract

##### Relationship of the fistulous tract with the sphincter complex

The description of the tract of a perianal fistula in correlation with the anatomy of the sphincter complex is of great relevance for surgical planning and should be done according to one of the classification systems, such as the Parks or St. James’s University Hospital classification^**(^2^)**^. The following aspects should be reported:

- The location and height at which the tract crosses the EAS
- The integrity of the IAS and EAS

##### Extension and secondary tracts

The presence of ramifications is significant when curative treatment is being considered, given that untreated ramifications increase the chance of recurrence. Secondary tracts, when present, should be reported in terms of their specific characteristics: anatomical location, whether cranial or caudal to the main tract; relationship with the levator ani (superelevator or infralevator); location and point of communication with the primary tract; and shape (blind-ended, horseshoe, etc.). Additional characteristics related to secondary tracts should be described, considering the following scenarios:

- **Horseshoe intersphincteric tract—**This is an important characteristic for planning fistulotomy, to predict the size of the wound; in the case of LIFT, it contraindicates the technique.
- **Angulated tract—**Complex fistulas with multiple ramifications or acute angulations are essential to report when sphincter-preserving procedures are being considered. These characteristics hinder evaluation with a rigid endoscope, increasing the risk of creating a false tract or making it difficult to identify secondary tracts in the case of VAAFT^**(^5^)**^. They are also limiting for FiLaC because of the difficulty of endoluminal access.
- **Extension of the tract—**This is an important characteristic in the case of long tracts. The results obtained with FiLaC seem to be better for tracts longer than 4 cm, given the shrinking effect that the laser has on the tissues around the laser fiber^**(^5^)**^.
- **Diameter of the tract—**Very narrow or very wide tracts are relevant. In the case of VAAFT, the fistula diameter must allow cannulation by the fistuloscope, which has a diameter of about 3.3 × 4.7 mm. In FiLaC, the laser acts to a depth at which the tract diameter is 2–3 mm, which reduces the risk of sphincteric injury but is less effective in fistulas with a diameter of 4–5 mm or greater^**(^5^)**^. In LIFT, the tract diameter is relevant for deciding which segment will be dissected and ligated. Tracts wider than 6 mm with an oblique or horseshoe tract limit the performance of the LIFT technique^**(^5^)**^.

##### External opening

The radial location of the external opening should be described according to the anal clock, and the anatomical location should be reported by region, such as gluteal, labial, etc.^**(^23^)**^. In addition, it is suggested that the distance between the external opening and the anal margin be measured and reported because, when it is long, it can result in a large surgical wound after fistulotomy.

##### Fluid collections

Report the presence or absence of collections. When they are present, report their characteristics, as follows:

- The anatomical location should be described by region (e.g., ischioanal fossa, perianal, etc.), and the radial location should be reported according to the anal clock.
- The size (diameter) should be reported as small (3– 10mm, not including the tract), medium (11–20 mm), or large (>20mm). If there are extensive collections, that fact should be promptly communicated to the referring team.
- Report whether there is a connection between the fluid collection and the main tract.

##### Other features

If present, the following features should also be commented upon:

- The inflammatory activity of the fistula can be reflected by several biomarkers on imaging, including a hyperintense signal on T2-weighted imaging and gadolinium contrast enhancement. Knowledge of that activity is beneficial in patients with Crohn’s disease who are undergoing drug therapy or seton drainage^**(^23^)**^. Although a healed tract may be seen on imaging after clinical improvement, this finding at any stage is a predictor of a favorable outcome—with a longer time free of perianal events, fewer hospitalizations, and fewer perianal surgeries— and should be included in the report.
- The presence of proctitis should also be reported, because it may limit the performance of rectal advancement flaps in the closure of the internal opening^**(^23^)**^.

#### 3D modeling

##### Overview

In the evaluation of perianal fistulas, 3D modeling from MRI data represents a novel and increasingly valuable adjunct. By translating conventional 2D sequences into interactive volumetric reconstructions, 3D models improve spatial understanding of complex fistulous anatomy, particularly regarding the relationship of tracts to the sphincter complex and pelvic floor structures. This enhanced visualization has been shown to facilitate communication between radiologists and surgeons, support multidisciplinary decision-making, and provide surgeons with a more intuitive roadmap for preoperative planning^**(^3,24,25^)**^.Notably, several studies have demonstrated that surgeons frequently reconsider the extent of disease or modify their operative strategy after reviewing 3D reconstructions in conjunction with standard MRI scans^**(^3,4,26^)**^.

##### Workflow and technical considerations

The generation of 3D fistula models typically begins with a standard pelvic MRI protocol. The backbone of that protocol is composed of conventional highresolution T2-weighted sequences in axial, coronal, and sagittal planes, often complemented by oblique sequences aligned with the anal canal. Some studies have incorporated isotropic 3D T2-weighted turbo spinecho sequences, which provide thin-slice coverage and allow multiplanar reconstructions with no gaps^**(^25,27^)**^. Segmentation of the fistula tract and relevant structures (internal and external sphincters, levator plate, perianal skin, and any abscesses or setons) is performed with semi-automated or manual tools. The various platforms include the Vitrea workstation (Vital Images, Minnetonka, MN, USA), as reported by Day et al.^**(^24^)**^, the Advantage workstation (GE Healthcare, Milwaukee, WI, USA), as reported by Lam et al.^**(^25^)**^, the interactive image visualization and segmentation tool ITK-SNAP (https://www.itksnap.org/pmwiki/pmwiki.php), combined with MeshLab (https://www.meshlab.net/), as reported by Sahnan et al.^**(^4^)**^, and dedicated medical modeling software packages such as Mimics (Materialise, Leuven, Belgium) and MIM (MIM Software Inc., Cleveland, OH, USA), which are used for segmentation, fusion, and 3D rendering of perianal fistula MRI datasets^**(^27^)**^. The development of the 3D model increases the report preparation time, with reported times for model development ranging from 20 min to 2 h^**(^26,27^)**^. Final models can be exported in stereolithography format, for 3D printing, or in interactive portable document format, for digital sharing and intraoperative consultation^**(^24,27^)**^. The 3D models illustrated in this review were generated from pelvic MRI datasets acquired in 1.5-T scanners, using the standard perianal fistula protocol of our institution. The imaging protocol included highresolution T2-weighted turbo spin-echo sequences in the axial, coronal, and sagittal planes, supplemented by contrast-enhanced T1-weighted imaging. For complex fistulas, an additional isotropic 3D turbo-spin-echo T2-weighted sequence without fat suppression was obtained. After standard interpretation of the 2D MRI scans by an experienced abdominal radiologist, manual segmentation was performed on the sequence that best demonstrated the fistulous tract and anatomic landmarks—most often the oblique axial T2-weighted or the contrast-enhanced T1-weighted sequence. The Digital Imaging and Communications in Medicine (DICOM) datasets were imported into ITK-SNAP, version 4.4.0, open-source software validated for 3D medical image segmentation. Each relevant structure was segmented in a distinct color to optimize spatial comprehension by surgeons: the fistulous tract (red), the IAS (green), the puborectalis muscle (light blue), and the EAS (dark blue).

##### Clinical integration

Across studies, the integration of 3D models into surgical planning has demonstrated several advantages. First, it improves diagnostic confidence, particularly in delineating secondary extensions, supralevator disease, and horseshoe fistulas^**(^3^)**^. Second, some software packages allow surgeons to rotate and zoom images in order to interact with the anatomy in real time, which enhances intraoperative orientation^**(^25^)**^. Third, in Crohn’s disease, 3D modeling has been used not only for surgical planning but also for monitoring responses to medical or surgical therapy, providing a reproducible visual tool for longitudinal follow-up^**(^4,25^)**^. Smith et al.^**(^26^)**^ reported that the use of 3D MRI reconstructions and corresponding physical models led to a change in the anatomical interpretation in 50% of fistula assessments. These findings support the notion that 3D modeling may reduce operative time, prevent incomplete sepsis drainage, and ultimately decrease recurrence rates^**(^3^)**^.

##### Limitations and future directions

Despite its promise, 3D modeling is not yet widely adopted in routine practice. The main limitations include segmentation challenges due to poor contrast between adjacent soft tissues, the need for radiologist expertise, additional postprocessing time, and software costs^**(^4,27^)**^. Printing physical models, although useful for education and patient communication, adds further expense and does not employ materials that would replicate softtissue properties^**(^11^)**^. In addition, the cost–benefit ratio for 3D modeling appears favorable when considering the reduction in recurrence and reoperation rates associated with improved preoperative mapping^**(^3^)**^. Future directions include streamlining segmentation through artificial intelligence, integrating 3D models into intraoperative navigation systems, and establishing standardized protocols to ensure reproducibility across centers.

## CONCLUSION

The use of MRI plays a pivotal role in the management of perianal fistulas, providing critical anatomical details that inform surgical strategy, reduce recurrence rates, and minimize complications. This review has synthesized current knowledge on optimizing MRI utilization, from conventional image acquisition and reporting to the emerging applications of 3D modeling.

## Data Availability

Not applicable
